# Georgia State Dental Society

**Published:** 1870-02

**Authors:** Arthur C. Ford

**Affiliations:** Recording Secretary.


### ARTICLE IV.

### *Georgia State Dental Society.*

By Arthur C. Ford, Recording Secretary.

Pursuant to the adjournment of the preliminary meeting of the Georgia State Dental Society, held in the city of Atlanta on the 30th of July last, it re-assembled in the Library Rooms of the Historical Society of Savannah at ten o'clock, A.M. President Dr. W. H. Burr, of Madison, in the chair.

Present, Dr. J. P. H. Brown, Cor. Sec., *Augusta;* F. Y. Clark, *Savannah;* H. A. Lowrence, *Athens;* E. M. Allen, *Marietta;* E. Parsons, *Sandersville;* W. Johnson, *Savannah;* A. M. Postley, *Savannah;* H. I. Royall, *Savannah;* E. L'Engle, *Savannah;* C. A. Harley, *South Carolina;* A. C. Ford, *Atlanta;* Mr. Samuel Hape, *Atlanta.*

In the absence of the Recording Secretary, Dr. T. J. Crow, of Macon, Arthur C. Ford was called upon to act pro tem.

The minutes of the preliminary meeting being then read, were slighly amended and confirmed.

Dr. J. P. H. Brown, one of the committee appointed to draft a Constitution and By Laws for the Society, then submitted a report, which on being read by sections, was somewhat amended and adopted, and signed by all the members present, and two hundred copies were ordered to be printed. The Society then adjourned to meet at 7.30 P.M. of the same day for the election of officers for the ensuing year.

December 28th, 7.30 P.M.

Society met agreeable to adjournment of this morning.

On motion of Dr. F. Y. Clark, P. P. Lewis, of Fla. and C. A. Harley, of S. C., (being present), were elected corresponding members, and Mr. Sam'l Hape, of Atlanta, Geo., an honorary member.

Dr. U. Van Guisen, of Geo., being present, signed the Constitution as an active member.

The Society then proceeded to the election of officers, with the following result:

| | | | |
|---|---|---|---|
| President, | Dr. | F. Y. Clark, | *Savannah.* |
| 1st Vice " | " | E. M. Allen, | *Marietta.* |
| 2nd " " | " | H. A. Lowrance, | *Athens.* |
| Cor. Secretary, | " | J. P. H. Brown, | *Augusta.* |
| Rec. " | " | A. C. Ford, | *Atlanta.* |
| Treasurer, | " | W. Johnson, | *Savannah.* |

*Executive Committee.*

Drs. Burr, Parsons, Allen, Lowrance and Johnson.

Society then adjourned to meet at 9 a.m. tomorrow.

December 29th, 9 a.m.

Agreeable to adjournment the Society met. Minutes of yesterday read and approved. Dr. F. Y. Clark, the President elect, was then installed in the chair, and the other officers assumed their duties.

The various committees were then appointed by the President, as follows:

*On Operative Dentistry:*—Drs. Parsons, Johnson, and Ford.
*On Mechanical Dentistry:*— Drs. Van Guisen, Royall, and Postley.
*On Dental Education:*—Drs. Brown, Holland, and Allen.

It was then moved by Dr. Brown that delegates be appointed to the Southern Dental Association and the American Dental Association; carried and the following delegates appointed:

*To the Southern Dental Association.*—Drs. Holland, Lewis, Johnson, Burr, Royall and Mr Sam'l Hape.

*To the American Dental Association.*—Drs. Allen, Brown, Ford, Van Guisen, Lowrance and Mr. Sam'l Hape.

A resolution was then offered by Dr, Burr as follows; which was adopted:

*Resolved*:—That this Society instruct their Delegates to the American and Southern Dental Associations, to request said Associations to appoint committees to petition Congress to appoint Dentists in the army and navy of the U. S.

An interesting and instructive essay was then read, and received, by Dr. F. Y. Clark on "The Diseases and Treatment of the Dental Pulp," and the subject was then opened to discussion, and several members gave their modes of treatment, experience, &c.

A. C. Ford moved,that a committee be appointed to prepare a paper upon "The Care and General Treatment of the Teeth *for the People*," to be submitted to the Society at its next annual session, subject to their approval, for publication ; adopted and Drs. Brown, Holland and Ford appointed.

Various other committees were then appointed for local purposes, and the Society adjourned to meet at 7.30 P.M.

DECEMBER 29TH, 7.30 P.M.

Society met agreeable to adjournment. Dr. Holland, of Augusta, being present signed the Constitution.

Moved by Dr. Allen that the Society now designate the place and time for the next session, carried. After some discussion, Atlanta, Geo., was selected, and the time Thursday, July 28th, 1870.

Moved by Dr. Allen that the Recording Secretary be instructed to furnish the *American Journal of Dental Science* with a synopsis of the proceedings of this Society, carried.

The President appointed Dr. Ford and Mr. Sam'l Hape, as committee of arrangements for next session.

After some further interchange of opinions, &c., on dental subjects, the Society then adjourned to meet in Atlanta, Geo., on Thursday, July 28th, 1870, at 10 o'clock, A. M.

---

